# Relationships between Volunteering, Neighbourhood Deprivation and Mental Wellbeing across Four British Birth Cohorts: Evidence from 10 Years of the UK Household Longitudinal Study

**DOI:** 10.3390/ijerph19031531

**Published:** 2022-01-29

**Authors:** Hei Wan Mak, Rory Coulter, Daisy Fancourt

**Affiliations:** 1 Department of Behavioural Science and Health, University College London, London WC1E 7HB, UK; hei.mak@ucl.ac.uk; 2 Department of Geography, University College London, London WC1E 6BT, UK; r.coulter@ucl.ac.uk

**Keywords:** volunteering, deprivation, cohorts, panel data analysis

## Abstract

Volunteering is associated with greater mental, physical and social wellbeing. However, less is known about whether the health benefits of volunteering vary with two sets of factors known to shape population health and health-related behaviours: (1) age and birth cohort, and (2) place of residence. This study examined how these factors influence the relationship between volunteering and self-reported mental health using five waves of data from Understanding Society: The UK Household Longitudinal Study (UKHLS) enriched with information on neighbourhood deprivation (Index of Multiple Deprivation 2015). Two self-reported mental health and wellbeing outcomes were examined: mental distress (GHQ-12) and health-related quality of life (SF-12). The sample was stratified by cohort: pre-1945 (born before 1945), Baby Boomers (born 1945–1964), Gen X (born 1965–1979), and Millennials (born from 1980). Fixed-effects regressions revealed that volunteering was associated with reduced levels of mental distress and greater levels of health-related quality of life in older generations, but not amongst younger generations. No moderating effect of area deprivation was found. This study suggests that generational social attitudes and changes in how volunteering is portrayed and delivered could influence not only whether people volunteer, but also whether doing so bolsters health.

## 1. Introduction

Volunteering—defined as individuals giving their time and labour voluntarily for community service—is recognised as an important asset for wellbeing [1]. Volunteerism spans a wide variety of both online and offline activities ranging from supporting neighbours with everyday tasks through to volunteering at a local library, animal shelter or community centre; teaching people about voting registration; working to improve the local environment (e.g., through litter picks); raising funds for charitable causes; involvement in local politics; and providing emotional support through helpline services. These activities vary in scale and formality from small, informal individual actions through to participating regularly in legally recognised community, national and global organisations. Globally, more than 1 billion people are thought to volunteer and in Britain, approximately 2 in 5 adults were estimated to have undertaken voluntary work in 2019 [2,3]. The scale of UK volunteering increased during the COVID-19 pandemic as more than 3000 self-organised mutual aid groups formed in the first months of 2020 [4]. Meanwhile, over 1 million people registered as volunteers with organisations such as the Royal Voluntary Service [5]. However, this rise might have subsided when social restrictions began to relax.

Engagement in voluntary work provides wide-ranging benefits for individual mental and social wellbeing [3,6,7]. A report from the National Council for Voluntary Organisations (NCVO) showed that 96% of volunteers (*N* = 10,103) reported feeling happy with the experience, with 9 in 10 reporting that volunteering provided enjoyment, a sense of personal achievement and purpose [3]. Furthermore, approximately 7 in 10 respondents reported improvements in their levels of loneliness and their overall mental health and wellbeing [3]. Results from the survey were in line with intervention studies, which showed that participants in the volunteering treatment group were more likely to experience improvements in life satisfaction, purpose in life, and personal growth [8], and were likelier to report fewer depressive symptoms [9]. Engaging in voluntary work is also associated with improved physical health. Reported physical benefits include decreased rates of functional decline, reduced cortisol reactivity to stressors and lower cardiovascular risk; as well as increased physical activity, strength and walking speed [6,10,11,12]. Volunteering also offers opportunities to widen social networks, enhance social bonds and connectedness, and it may provide social rewards [6,13,14].

While the health benefits of volunteering are well documented in both experimental and cohort studies, there has been growing interest in understanding the *mechanisms* producing this relationship. A recent rapid evidence assessment identified nine mechanisms through which volunteering promotes health: social connections; appreciation; sense of purpose and meaning; skills and knowledge development; role and group identity; feeling of enjoyment; structure and routine; exposure to the outdoors and nature; and clear and low intensity of voluntary work demands [15]. For instance, volunteering provides opportunities to build new social relationships and connections with others, which are particularly beneficial for individuals experiencing a loss of social networks through life changes (e.g., retirement or family transitions such as separation) [16]. Interestingly, the impacts of volunteering are stronger when volunteers feel appreciated, with a recent UK longitudinal study showing that reciprocity (i.e., receiving adequate appreciation from others) in voluntary work is positively associated with quality of life and decreased odds of depression for both men and women [17]. This finding could be explained by the reward obtained from appreciation that may, in part, reflect the social value accruing from volunteering [17]. In addition, volunteering activity provides a sense of altruistic purpose which is linked with better wellbeing [18].

Yet while previous research has established that volunteering is broadly associated with improved wellbeing, far less is known about whether the health benefits of volunteering vary across the population with (i) age and birth cohort and (ii) place of residence. Both factors are known to shape population health and the literature hints they may also moderate how volunteering impacts wellbeing. We now turn to discuss each of these potential moderators in turn.

### 1.1. Age and Birth Cohort

On the one hand, there is some evidence that volunteering improves health across the life course. Studies of older adults show that taking part in voluntary work is associated with reduced depression as well as higher levels of life satisfaction, self-esteem, and social support [6]. Volunteering also appears to mitigate the negative effects of low self-esteem on sense of belonging and life satisfaction [19]. For younger adults aged approximately 18–24, volunteering is related to reduced loneliness and increased confidence [3]. Furthermore, youth studies have suggested that engagement in voluntary work is associated with lower risk of school truancy and that it facilitates moral development and encourages socially responsible actions [20].

On the other hand, some evidence indicates that the positive impacts of volunteering are greater for older adults. For example, a systematic review of 40 papers using experimental and observational data reported that older volunteers may be more likely to experience reduced functional dependency and fewer depressive symptoms than younger people [12]. Meanwhile, a life-course analysis showed that the association between volunteering and wellbeing emerges when individuals reach 40 years, before continuing through to old age [21]. A possible explanation could be that older volunteers may simply have engaged in voluntary work for longer and/or at a greater frequency (e.g., due to having extra time after retirement) and hence have reaped greater positive impacts from their engagements by mid- and later life [22]. It is also plausible that younger adults have stronger social connections at home (for example when raising children) and/or in the workplace than older [23], retired adults who have fewer everyday contacts and thus may depend more on opportunities such as volunteering to meet people. As a result, dependence on volunteering may be higher as age increases.

However, there may not be a linear relationship between time spent on volunteering and wellbeing. Research shows that heavy volunteering commitments—rather like heavy care burdens—could have adverse effects on health and wellbeing [12]. Furthermore, the benefits of volunteering seem to emerge with only small amounts of engagement [3]. The latter point hints that differences in frequency or intensity of engagements may not fully explain why older adults generally enjoy greater health and wellbeing benefits from volunteering. It is also important to recognise that as age rises, survivor bias in many studies increases as less healthy adults selectively exit the population through death. This suggests that older volunteers may be a more selectively healthy group than younger volunteers. Addressing this issue requires using longitudinal data and fixed-effects methods to control for unobserved (and possibly unobservable) baseline differences between those volunteering at different points in the life course.

Age differences in the health benefits of volunteering may also be confounded by cohort effects derived from differences in the way the social meaning of volunteering has changed through time. In the twentieth century, volunteerism expanded dramatically during the World Wars and boomed in the 1960s when there was a strong collective ethos and when society was becoming more economically equal [24,25]. While volunteering continued to be promoted from the 1980s, [24,26,27,28], the spread of individualism, as well as significant technological advancements (in particular online communications and most recently social media) may mean that the social benefits of volunteering have declined. Relatedly, a rise in reported mental health conditions in recent generations may suggest that people in younger cohorts who engage in voluntary work experience poorer baseline mental health, which could alter how volunteering impacts on their wellbeing [29]. Indeed, volunteering is increasingly promoted in a more instrumental fashion to support individual health and wellbeing. This is reflected in current UK social prescribing schemes where volunteering is positioned as an activity that has the potential to improve social, mental, and physical wellbeing [30]. Thus, more nuanced work exploring both age and cohort effects of volunteering for physical and mental health is needed.

### 1.2. Neighbourhoods

Although most research into the health payoff of volunteering concentrates on individual-level predictors, the public health literature comprehensively demonstrates that both health and healthy behaviours vary across places and between neighbourhoods in particular [31]. Policy makers have long assumed neighbourhoods matter for civic engagement and health and in Britain, the government has launched place-based funding streams and programmes to promote local community activities such as volunteering and charity work [27,30].

However, relatively little is known about whether volunteering and especially its health benefits vary across neighbourhoods. A US study showed that perceived neighbourhood safety was positively correlated with volunteering, possibly due to a greater likelihood of leaving home and engaging in social activities [32]. Similarly, neighbourhoods with stronger sense of community, connectedness, and with more community amenities and services provide greater incentives for adults to participate in volunteer activities [33,34]. The nature of the built environment may also influence volunteering. For example, one study found that adults with lower levels of education were more likely to perceive transportation as a barrier to engagement, although this effect was, perhaps unsurprisingly, stronger in less connected rural areas [35]. In England, there is some evidence that volunteering tended to be lower in more deprived areas and in areas with fewer charities [36,37]. Understanding how the impact of volunteering may vary geographically is particularly timely with the current UK government’s “levelling up” agenda, given that volunteers could help create stronger communities and yet they may be spread unevenly across areas and may be concentrated away from more deprived areas where they are most needed.

In view of the above, this study sought to examine the longitudinal association between volunteering and mental distress (GHQ-12) and health-related quality of life (SF-12) over a 10-year follow-up. To test whether the association varies across birth cohorts, the sample was stratified by cohort: pre-1945 (born before 1945), Baby Boomers (those born in 1945–1964), Gen X (born in 1965–1979), and Millennials (born from 1980). To test whether the association varies geographically with area deprivation, interactions between volunteering and area deprivation measured using the Index of Multiple Deprivation (IMD) were included in analyses.

## 2. Data and Methods

Data came from Understanding Society: The UK Household Longitudinal Study (UKHLS). The UKHLS follows over 50,000 individuals from 30,000 households annually [38] and collects rich information on participants’ socio-demographics, community group participation and volunteering engagement, as well as their mental health and wellbeing. In this study, we extracted a sample of adults living in England who responded in waves 2 (2010/12; response rate = 84%), 4 (2012/14; response rate = 84%), 6 (2014/16; response rate = 84%), 8 (2016/18; response rate = 88%), and 10 (2018/20; response rate = 88%), where volunteering engagement was measured. The proportion of participants completing at least one of these five waves was 81%. We only considered respondents providing data across all measures (number of person-year observations = 128,736; number of individuals = 40,998), as well as respondents with a valid longitudinal weight. This left an analytical sample of 51,206 observations from 10,989 participants (4.7 per person, ranging from 1 to 5). The University of Essex Ethics Committee approved the UKHLS and participants provided informed consent.

To investigate the role of neighbourhood deprivation, we used geo-coded UKHLS data in which participating households’ addresses were matched to neighbourhood zones. Neighbourhoods were defined as 2011 census Lower Super Output Areas (LSOAs) [39]. In the 2011 census there were 32,844 LSOAs in England with populations ranging from 1000 to 3000 (mean = 1614) [39,40].

### 2.1. Measures

*Index of Multiple Deprivation* (*IMD*): Using the 2011 LSOA geocodes, we attached the 2015 English Index of Multiple Deprivation decile (IMD, 2015). IMD uses a range of input datasets to rank the relative deprivation of LSOAs across seven weighted domains: income, employment, health deprivation and disability, education, skills and training, crime, barriers to housing and services, and living environment [41]. In our main analysis, a binary variable was generated for each wave to indicate whether respondents lived in an LSOA classed as one of the 20% most deprived in England (the most deprived quintile). Three alternative specifications were generated for sensitivity checks: (i) a binary variable with a more stringent deprivation threshold indicating whether respondents lived in an LSOA classed as in the most deprived 10% (decile); (ii) a set of seven binary variables (using the 20% most deprived as the threshold) created for each IMD domain [42]; (iii) and a continuous variable using LSOA IMD rank scores (ranging from 1 to 32,829 for the analytical sample) for a more nuanced moderator. Results of these sensitivity checks are discussed below.

*Volunteering*: Respondents were asked how often they had given any unpaid help or worked as a volunteer for any type of local, national or international organisation or charity in the last 12 months. Frequency of engagement was categorised as “not in the last 12 months”, “helped or worked on a seasonal basis”, “one-off activity”, “just a few times”, “quite often but not regularly”, “at least once a month”, “once a fortnight”, “once a week”, “twice a week”, and “on 3 or more days a week”. In our sample, 79% did not engage in voluntary work, 6.2% engaged infrequently (either one-off activity, just a few times, or not regularly), and 15% engaged at least once a month. In the main analysis, a binary variable was created (1 = engaged in any levels of volunteering, 0 = did not volunteer). Approximately 37.6% of our sample had changed the binary category of volunteering. For sensitivity analysis, we additionally generated a binary variable that used a higher frequency of engagement as the threshold with which to identify volunteers (1 = engaged at least once a month, 0 = engaged less than once a month or none). Results of the sensitivity analysis are discussed below.

*Mental distress* (*GHQ-12*): Mental distress was measured using the GHQ-12 (General Health Questionnaire), a screening device designed to identify psychiatric disorders in the general population and in primary medical care settings [43,44]. The GHQ-12 self-reported questionnaire includes 12 4-point items (such as sleeping problems, overall happiness and depressive symptoms, α = 0.90). All scores were summed and averaged with a scale of 1 to 4. Higher scores indicate greater levels of mental distress.

*Health-related quality of life* (*SF-12*): The SF-12 survey (12-Item Short Form Health Survey; α = 90) has been designed to measure health-related quality of life. It includes 12 items and consists of eight components: general health, physical functioning, role physical, body pain, social functioning, role emotional, mental health, and vitality [45] (α = 0.90). All scores were summed and averaged with a scale of 1 to 5. Higher scores indicate greater levels of health-related quality of life.

*Time-varying covariates*: Ten time-varying variables that might confound observational associations between volunteering and health were identified for the analysis. These included *demographic characteristics*: age, partnership status (married/in cohabitation vs. not married/not in cohabitation), living with parents (yes vs. no), living with children (yes vs. no), number of close friends, and long-standing limiting illness or impairment (yes vs. no); *socio-economic position* (*SEP*): education (degree vs. no degree), employment status (employed vs. not employed), and individual monthly income; and *level of area deprivation* (LSOA in the 20% most deprived vs. not in the 20% most deprived).

### 2.2. Analysis

Data were analysed using fixed-effects regression, a panel data method which controls for all time-invariant (un)observed variables [46]. As fixed-effects models use only within-individual variation, they are more robust than between-unit models (e.g., traditional regressions) when examining the relationship between changes in volunteering engagement and changes in mental health and wellbeing.

In the main analyses, fixed-effects models were fitted separately for the two outcome variables: mental distress (GHQ-12) and health-related quality of life (SF-12). Each model was built sequentially: Model 1 controlled for volunteering engagement, Model 2 additionally adjusted for demographic background and SEP, and Model 3 additionally controlled for area deprivation. To test whether the association between volunteering and mental health and wellbeing varied with neighbourhood deprivation, moderation analysis was conducted individually using level of area deprivation as a moderator. All analyses were carried out using Stata/SE 17.0.

## 3. Results

### 3.1. Descriptive

In the entire sample, the overall mean for volunteering was 0.21 (indicating 21% of our sample volunteered with different levels of frequency), with a greater prevalence of volunteering among older cohorts. The overall means for mental distress and health-related quality of life were 1.92 and 3.77, respectively. Younger cohorts had higher means for both outcomes (Table 1), indicating worse mental distress but better health-related quality of life.

**Figure 1:**
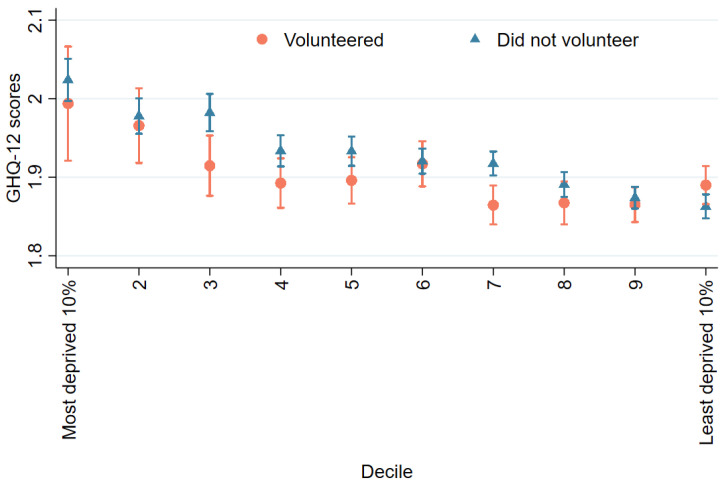
Mental distress (GHQ-12).

**Figure 2:**
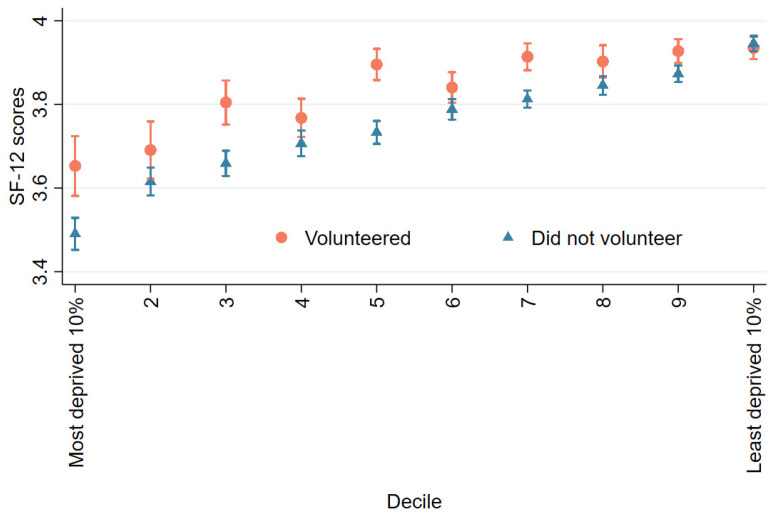
Health-related quality of life (SF-12).

When exploring the correlations between volunteering, mental health/wellbeing, and area of deprivation, cross-tabulation shows that volunteers generally experienced lower levels of mental distress and greater levels of health-related quality of life than non-volunteers. These differences were more prominent in more deprived neighbourhoods for SF-12. Levels of mental distress and health-related quality of life were worse in more deprived areas for both volunteers and non-volunteers (Figure 1 and Figure 2).

### 3.2. Mental Distress (GHQ-12)

**Figure 3:**
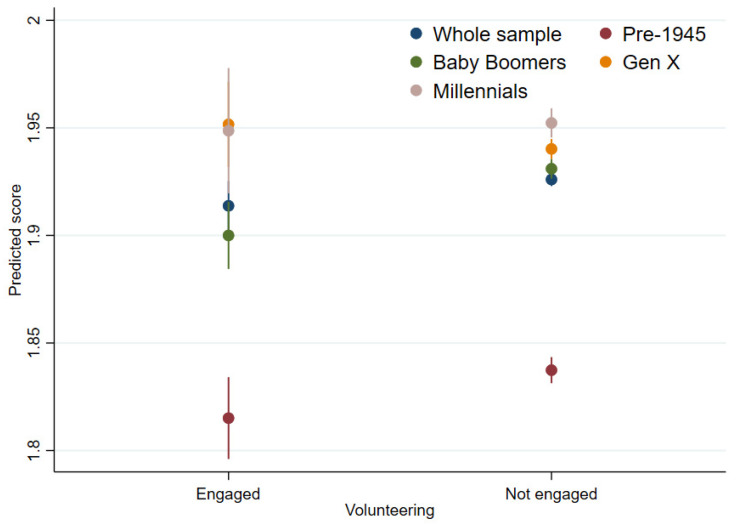
Volunteering and mental distress (GHQ-12).

Fixed-effects regressions (Model 3; Table 2 and Figure 3) did not show an overall association between changes in volunteering and changes in mental distress when looking at all age cohorts together. However, it did show that volunteering was associated with lower levels of mental distress for the Baby Boomer generation specifically (coef = −0.03, 95%CI = −0.05, −0.01). The negative association was also seen for the pre-1945 generation (Model 1; Table 2), but the association was less definite and became insignificant altogether after controlling for time-varying confounders (coef = −0.03, 95%CI = −0.05, 0.00). No associations were shown in the full sample nor for the Gen X or Millennials generations (Model 3; Table 2). No moderating effect of area deprivation was shown for mental distress (Table 3).

### 3.3. Health-Related Quality of Life (SF-12)

**Figure 4:**
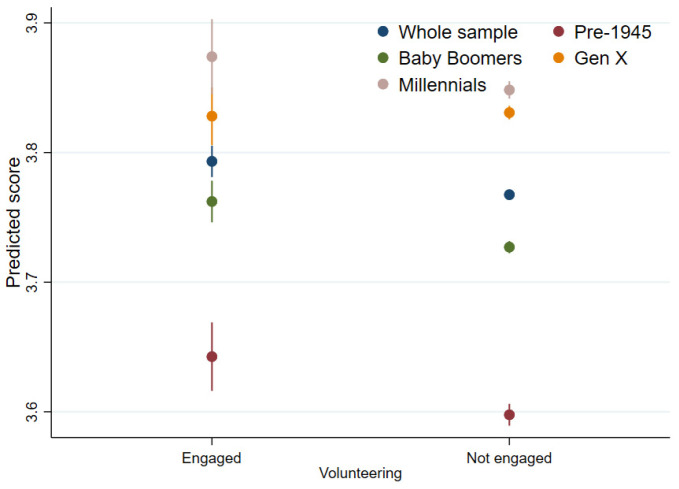
Volunteering and health-related quality of life (SF-12).

For health-related quality of life (Model 3; Table 4 and Figure 4), volunteering was associated with greater levels of quality of life amongst the sample as a whole (coef = 0.03, 95%CI = 0.01, 0.04). When looking at specific age cohorts, positive associations were shown for the pre-1945 (coef = 0.04, 95%CI = 0.01, 0.08) and Baby Boomers (coef = 0.04, 95%CI = 0.01, 0.06) generations. In Model, 1 there was also some indication that volunteering engagement was related to higher levels of quality of life amongst the Millennials (coef = 0.04, 95%CI = 0.00, 0.08); however, the association was attenuated after time-varying confounders were added (Table 4; Figure 4). No moderating effect of area deprivation was shown (Table 5).

### 3.4. Sensitivity Analysis

For our first sensitivity analysis, we ran the models adjusting for age variable only and found that the associations between changes in volunteering and changes in the outcomes were attenuated once age was controlled for (Table S1).

Next, we reran all models comparing respondents who volunteered at least once a month to those who volunteered less or did not volunteer. Results are very similar to those in Table 1 and Table 2 with older generations experiencing positive mental health and wellbeing through volunteering (Tables S2 and S3).

Then, we used a more extreme threshold (i.e., 10% most deprived neighbourhoods vs. 90% less deprived) to test the role of area deprivation. Results were almost identical to those when using the 20% threshold and no interaction effects were shown (Table S4).

We also repeated the interaction models, but this time we separated each of the seven domains of IMD to tested whether different aspects of deprivation had different moderating effects (coefficients and 95%CIs were shown in Figures S1 and S2). Here, the results showed some interaction effects for the Millennials sample. In particular, stronger effects of volunteering on reduced mental distress were shown in the 20% most employment deprived areas (less deprived: coef = 0.02, 95%CI = −0.02, 0.05; 20% most deprived: coef = −0.08, 95%CI = −0.16, 0.00). We also found that the impacts of volunteering on health-related quality of life were more salient in areas of high employment deprivation (less deprived: coef = 0.00, 95%CI = −0.03, 0.04; 20% most deprived: coef = 0.12, 95%CI = 0.01, 0.22) and in the 20% most health deprived areas (less deprived: coef = 0.00, 95%CI = −0.03, 0.04; 20% most deprived: coef = 0.13, 95%CI = 0.03, 0.23).

Finally, we used the rank of IMD to test the interaction effects; no effects were shown (Table S5).

## 4. Discussion

This study examined the longitudinal association between volunteering and mental health and wellbeing over a 10 year follow up. Fixed-effects analysis revealed that increasing one’s volunteering is associated with greater levels of health-related quality of life amongst adults as a whole, but these effects are largely driven by older generations (Baby Boomers and adults born before 1945). Baby Boomers also appear to benefit more from volunteering as a means of reducing mental distress. There was little evidence that younger adults born from 1965 onwards experience the same outcome from volunteering, although preliminary evidence suggests that Millennials living in areas of greater employment deprivation and health deprivation may experience higher levels of health-related quality of life from volunteering (although sample size limitations mean this result should be treated cautiously). Further, we found no moderating effect of area deprivation on the association between volunteering and mental health and wellbeing.

Our findings on health-related quality of life are in line with previous research documenting an association between engagement in voluntary work and improved positive wellbeing [3,6,10,11,12,19,47,48]. In particular, we found that people in older generations were more likely to experience these positive impacts, and that the association was not moderated by where they lived. This can be understood through the lens of age and cohort effects. Age effects are found when changes in physical, social, mental, and behavioural experiences are linked to aging, but unrelated to the birth cohort to which individuals belong. Indeed, there is plenty of research showing the effects of volunteering on the wellbeing of individuals in later years of life [6,10,13,19,47], with evidence pointing to the stronger effects amongst older populations when compared to younger adults [12,21]. Previous research identified a number of mechanisms to understand *how* the experience of volunteering improves mental health and wellbeing: some of these mechanisms may be especially relevant for older adults [15], such as the new social networks developed through engagement [16], social role and group identity, and a provision of structure to volunteers’ lives [49]. In contrast, many key ingredients (e.g., personal development, relationships, values and identities) [15] that link volunteering to health and wellbeing for younger adults could be experienced through education, employment, and family responsibilities. This has been reflected in our analysis where the association between volunteering and health-related quality of life was attenuated after considering time-varying factors including age, changes in household composition and employment status.

The fact that the effects of volunteering vary across cohorts after controlling for age indicates that there are cohort effects on the health benefits of volunteering. Change over time in the social meaning of volunteering, alongside initiatives and programmes that encourage engagements, could potentially influence cohort motives, values and beliefs towards volunteering (i.e., how people think about their volunteer work), their voluntary behaviour, and the associated outcomes. In addition, the changes in social and political contexts could also influence volunteering engagement. For instance, lower incomes and reduced economic security may have made it challenging for adults in younger generations to commit to volunteering on a regular basis, or to be involved in voluntary activities within a neighbourhood [50]. Infrequent voluntary involvement may have weakened the benefits of engagements [22]. It is also possible that people who were born in different generations may have collective characteristics which in turn affect the rate of engagement and its associated outcomes. For instance, older generations such as the pre-1945 and Baby Boomers generations have been characterised as committed, disciplined and who enjoy direct and in person communications, as opposed to younger generations who grew up with digital communication technologies, may be more individualist, and are more likely to suffer from poor mental health [51,52]. As a result, it is possible that while older generations may get involved in volunteering to support society, younger generations may have used volunteering as a way to improve their wellbeing and support personal development. Different rationales for volunteering may influence its health impacts and this is an important area for further work.

Notably, whilst our findings show overall effects for health-related quality of life, they do not show for mental distress. This reflects the mental health continuum model, in which mental wellbeing and mental illness are both related but distinct and they are not opposite ends of a single continuum [53]. This suggests that volunteering may help support health-related quality of life (mental wellbeing) but may have less effect on alleviating mental distress (mental illness). One possible explanation for this is that such engagement may have a more direct effect on individuals’ physical and mental wellbeing due to the activities involved, such as physical activities (e.g., deliveries, walking, reduced sedentary behaviour) and interactions with other volunteers. In contrast, the benefits of voluntary engagement on mental distress may be more indirect (e.g., through an expansion of social networks and providing a more structured daily routine), and that a greater frequency of the engagement may be needed for the protective effects on mental distress to be detected (as suggested in the sensitivity check). Nonetheless, the effects on mental distress were found for the Baby Boomers generation. This is in line with a previous study [21], which found that the association between voluntary work and mental distress did not appear until the age of 40 years and continued up to old age (roughly corresponding to the age of the Baby Boomers generation in our sample).

Generally, we found no moderating effect of area deprivation, suggesting that the positive wellbeing associated with volunteering occurs regardless of where people live. The lack of a neighbourhood deprivation effect may reflect a more equal number of voluntary organisations in the least and most deprived areas [54] and a growth of place-based funding streams that encourage residents in deprived areas to engage in voluntary and community work (e.g., through creating safer neighbourhoods, ensuring well-maintained voluntary organisations, and providing wide ranging voluntary activities and flexible volunteering hours). However, there are other potential explanations too. First, it is possible that the lack of interaction effects might have been driven by the small main effects for area deprivation on the outcome measures. As a result, there might not have been a sufficient statistical power for ‘true’ interaction effects to be detected. Second, our findings might have been caused by a small effect of neighbourhood deprivation on the likelihood of volunteering. Indeed, whilst there were differences in volunteering engagement rate across different levels of area deprivation, only a small proportion of the variation in volunteering was attributed to variations between localities once individual characteristics had been accounted for [28]. Nonetheless, our findings have potentially important implications for the “levelling up” agenda as they suggest that volunteering could have benefits for all individuals if it can be introduced to different areas equally. To enable this, it is important to ensure the availability of the central grant funding, particularly, in more deprived local authorities where voluntary organisations are likely to face greater difficulty in obtaining income from their councils [55]. In addition, creating a safer, harmonic, and close-knit residential environment, coupled with neighbourhood stability (through affordable home ownership schemes), which help establish senses of place belonging and attachment, may also help improve the engagement rate in volunteering [23].

Our study has a number of strengths, including the large sample and the use of fixed-effects analysis to effectively control for all time-invariant variables as well as observed time-varying health predictors. However, this study is not without limitations. As this study examined parallel longitudinal associations between volunteering and mental health and wellbeing, causal inferences cannot definitively be established, although the findings are in line with experimental research [12]. Further, whilst we examined and compared associations across birth cohorts, there is still a risk of selection bias where the analysis for older generations only includes respondents who survived (i.e., people who were healthier physically and mentally). While the categorisation of the cohorts in the present study is used to align well with popularly recognised generations, there might still be a possibility that individuals from the same cohort experienced slightly different political and economic environments. Future work is also needed to explore the interactions between age, period and cohort effects.

In addition, in our moderating analysis, we only focused on neighbourhoods where people lived, instead of where they volunteered. Whilst people tend to volunteer within relatively local communities, it is also possible that some may travel to a different neighbourhood where more volunteering opportunities are more readily available. Finally, more studies are required to better assess whether the frequency of engagement as well as the variety of voluntary activities influence the impacts of volunteering on health and wellbeing. Previous studies have suggested that volunteers approaching their 50s were more interested in volunteering in connection with religious organisations, citizens’ groups, and caring for the elderly, whereas voluntary work relating to youth issues (e.g., improving children’s education) were more popular amongst younger volunteers aged between 25–44 [23]. More recently, social action volunteering around themes such as human migration, climate change, and race inequality may have attracted more younger volunteers. Types of voluntary work may result in appreciable variations in volunteers’ health and wellbeing and this is an important topic for further research.

## 5. Conclusions

There is a growing consensus that the wellbeing of individuals and communities with a strong sense of cooperation and compassion (core values of volunteerism) helps a society thrive [2]. This study shows that participating in volunteering is associated with higher levels of health-related quality of life and that the association can be found across neighbourhoods, regardless of deprivation. This is in line with previous studies. However, the present study goes beyond this to show that the effects of volunteering are greatest for people from older birth cohorts. Given that age is controlled in our analysis, this evidence suggests that generational social attitudes and changes in how volunteering is portrayed and delivered (e.g., as a means to improve collectively improve society vs. a strategy to promote individuals’ health and wellbeing) could influence not only whether people volunteer, but also whether doing so bolsters health. It is particularly encouraging that older volunteers are experiencing the positive impacts through volunteering as this suggests that promoting volunteer work to older adults could help the UK population to age healthily.

## Figures and Tables

**Table 1:** Descriptive statistics of volunteering frequency and mental health/wellbeing.

Descriptive Statistics of Volunteering Frequency and Mental Health/Wellbeing					
Variables of Interest	Whole Sample	Pre-1945 (Born before 1945)	Baby Boomers (Born in 1945–1964)	Gen X (Born in 1965–1979)	Millennials (Born in 1980 or After)
**Volunteering**					
Overall mean	0.21	0.24	0.23	0.19	0.19
Between-participant SD (σ_u_)	0.33	0.37	0.34	0.29	0.31
Within-participant SD (σ_e_)	0.30	0.28	0.29	0.31	0.33
Intraclass correlation (ρ)	0.54	0.63	0.58	0.47	0.47
**Mental distress (GHQ 12)**					
Overall mean	1.92	1.83	1.92	1.94	1.95
Between-participant SD (σ_u_)	0.34	0.27	0.34	0.33	0.38
Within-participant SD (σ_e_)	0.33	0.26	0.31	0.34	0.39
Intraclass correlation (ρ)	0.51	0.52	0.55	0.48	0.49
**Health-related quality of life (SF-12)**					
Overall mean	3.77	3.61	3.74	3.83	3.85
Between-participant SD (σ_u_)	0.55	0.56	0.60	0.51	0.47
Within-participant SD (σ_e_)	0.37	0.40	0.36	0.37	0.39
Intraclass correlation (ρ)	0.68	0.66	0.74	0.65	0.59
**Number of obs**	51,206	7351	21,809	13,256	8790
**Number of groups**	10,989	1491	4431	2702	2365

**Table 2:** Fixed-effects analysis predicting the associations between volunteering and mental distress (GHQ-12).

Fixed-Effects Analysis Predicting the Associations between Volunteering and Mental Distress (GHQ-12)											
Sample	Model 1 Volunteering			Model 2 = Model 1 + Demography and SEP			Model 3 = Model 2 + IMD			Number of Obs	Number of Groups
	Coef	95%CI	*p*-Value	Coef	95%CI	*p*-Value	Coef	95%CI	*p*-Value		
Whole sample	−0.01	−0.03, 0.00	0.121	−0.01	−0.03, 0.00	0.099	−0.01	−0.03, 0.00	0.101	51,206	10,989
Pre-1945 (born before 1945)	**−0.03**	**−0.05, −0.00**	**0.026**	−0.02	−0.05, 0.00	0.082	−0.02	−0.05, 0.00	0.082	7351	1491
Baby Boomers (born in 1945–1964)	**−0.03**	**−0.05, −0.01**	**0.007**	**−0.03**	**−0.05, −0.01**	**0.003**	**−0.03**	**−0.05, −0.01**	**0.003**	21,809	4431
Gen X (born in 1965–1979)	0.02	−0.01, 0.04	0.213	0.01	−0.01, 0.04	0.359	0.01	−0.01, 0.04	0.360	13,256	2702
Millennials (born in 1980 or after)	−0.01	−0.05, 0.03	0.592	−0.00	−0.04, 0.03	0.833	−0.00	−0.04, 0.03	0.844	8790	2365

Notes: All models controlled all time-invariant variables. Model 1 additionally included volunteering engagement. Model 2 = Model 1 + demography (age, partnership status, whether or not living with parents, whether or not living with children, number of close friends, and long-standing illness or impairment) and SEP (education levels, employment statues, and individual monthly income). Model 3 = Model 2 + IMD (whether or not living in the 20% most deprived areas). Bold values denote statistical significance at the *p* < 0.05 level.

**Table 3:** Fixed-effects models interacting with Index of Multiple Deprivation (IMD) 20% most deprived areas: Mental Distress (GHQ-12).

Fixed-Effects Models Interacting with Index of Multiple Deprivation (IMD) 20% Most Deprived Areas					
Sample	Mental Distress (GHQ-12)			Number of Obs	Number of Groups
	Coef	95%CI	*p*-Value		
**Whole sample**				51,206	10,989
Volunteering	−0.01	−0.03, 0.00	0.133		
IMD 20% most deprived	−0.03	−0.07, 0.02	0.281		
Volunteering * IMD 20% most deprived	−0.01	−0.06, 0.04	0.788		
**Pre-1945 (born before 1945)**				7351	1491
Volunteering	−0.02	−0.05, 0.00	0.100		
IMD 20% most deprived	−0.12	−0.30, 0.07	0.213		
Volunteering * IMD 20% most deprived	−0.00	−0.08, 0.08	0.999		
**Baby Boomers (born in 1945–1964)**				21,809	4431
Volunteering	**–0.03**	**–0.05, –0.01**	**0.001**		
IMD 20% most deprived	−0.02	−0.11, 0.08	0.732		
Volunteering * IMD 20% most deprived	0.02	−0.07, 0.11	0.736		
**Gen X (born in 1965–1979)**				13,256	2702
Volunteering	0.01	−0.02, 0.03	0.529		
IMD 20% most deprived	0.01	−0.06, 0.08	0.807		
Volunteering * IMD 20% most deprived	0.02	−0.06, 0.10	0.591		
**Millennials (born in 1980 or after)**				8790	2365
Volunteering	0.01	−0.03, 0.05	0.732		
IMD 20% most deprived	−0.02	−0.08, 0.05	0.674		
Volunteering * IMD 20% most deprived	−0.05	−0.14, 0.05	0.326		

Notes: All models controlled all variables shown in the in-text analysis. Bold values denote statistical significance at the *p* < 0.05 level. * symbol represents interaction terms.

**Table 4:** Fixed-effects analysis predicting the associations between volunteering and health-related quality of life (SF-12).

Fixed-Effects Analysis Predicting the Associations between Volunteering and Health-Related Quality of Life (SF-12)											
Sample	Model 1 Volunteering			Model 2 = Model 1 + Demography and SEP			Model 3 = Model 2 + IMD			Number of Obs	Number of Groups
	Coef	95%CI	*p*-Value	Coef	95%CI	*p*-Value	Coef	95%CI	*p*-Value		
Whole sample	**0.03**	**0.02, 0.05**	**0.000**	**0.03**	**0.01, 0.04**	**0.001**	**0.03**	**0.01, 0.04**	**0.001**	51,206	10,989
Pre-1945 (born before 1945)	**0.09**	**0.05, 0.13**	**0.000**	**0.04**	**0.01, 0.08**	**0.012**	**0.04**	**0.01, 0.08**	**0.012**	7351	1491
Baby Boomers (born in 1945–1964)	**0.03**	**0.01, 0.05**	**0.003**	**0.04**	**0.01, 0.06**	**0.001**	**0.04**	**0.01, 0.06**	**0.001**	21,809	4431
Gen X (born in 1965–1979)	−0.01	−0.04, 0.02	0.611	−0.00	−0.03, 0.02	0.852	−0.00	−0.03, 0.02	0.846	13,256	2702
Millennials (born in 1980 or after)	**0.04**	**0.00, 0.08**	**0.041**	0.03	−0.01, 0.06	0.157	0.03	−0.01, 0.06	0.159	8790	2365

Notes: All models controlled all time-invariant variables. Model 1 additionally included volunteering engagement. Model 2 = Model 1 + demography (age, partnership status, whether or not living with parents, whether or not living with children, number of close friends, and long-standing illness or impairment) and SEP (education levels, employment statues, and individual monthly income). Model 3 = Model 2 + IMD (whether or not living in the 20% most deprived areas). Bold values denote statistical significance at the *p* < 0.05 level.

**Table 5:** Fixed-effects models interacting with Index of Multiple Deprivation (IMD) 20% most deprived areas: Health-Related Quality of Life (SF-12).

Fixed-Effects Models Interacting with Index of Multiple Deprivation (IMD) 20% Most Deprived Areas					
Sample	Health-Related Quality of Life (SF-12)			Number of Obs	Number of Groups
	Coef	95%CI	*p*-Value		
**Whole sample**				51,206	10,989
Volunteering	**0.02**	**0.01, 0.04**	**0.004**		
IMD 20% most deprived	−0.00	−0.05, 0.05	0.973		
Volunteering * IMD 20% most deprived	0.02	−0.03, 0.08	0.375		
**Pre-1945 (born before 1945)**				7351	1491
Volunteering	**0.04**	**0.00, 0.07**	**0.035**		
IMD 20% most deprived	−0.07	−0.30, 0.16	0.547		
Volunteering * IMD 20% most deprived	0.08	−0.08, 0.23	0.335		
**Baby Boomers (born in 1945–1964)**				21,809	4431
Volunteering	**0.04**	**0.02, 0.06**	**0.000**		
IMD 20% most deprived	−0.08	−0.19, 0.03	0.142		
Volunteering * IMD 20% most deprived	−0.02	−0.11, 0.07	0.614		
**Gen X (born in 1965–1979)**				13,256	2702
Volunteering	0.00	−0.03, 0.03	0.908		
IMD 20% most deprived	0.05	−0.03, 0.13	0.193		
Volunteering * IMD 20% most deprived	−0.03	−0.11, 0.04	0.380		
**Millennials (born in 1980 or after)**				8790	2365
Volunteering	0.01	−0.03, 0.04	0.725		
IMD 20% most deprived	−0.01	−0.08, 0.07	0.888		
Volunteering * IMD 20% most deprived	0.09	−0.01, 0.19	0.091		

Notes: All models controlled all variables shown in the in-text analysis. Bold values denote statistical significance at the *p* < 0.05 level. * symbol represents interaction terms.

## Data Availability

UKHLS data are available from the UK Data Service. Understanding Society: Waves 1–10, 2009–2019 and Harmonised BHPS: Waves 1–18, 1991–2009 are available at https://beta.ukdataservice.ac.uk/datacatalogue/studies/study?id=6614 (accessed on 8 December 2020); Understanding Society: Waves 1–10, 2009–2019: Special Licence Access, Census 2011 Lower Layer Super Output Areas are available at https://beta.ukdataservice.ac.uk/datacatalogue/studies/study?id=7248 (accessed on 7 October 2021); Index of Multiple Deprivation data can be obtained from https://www.gov.uk/government/statistics/english-indices-of-deprivation-2015 (accessed on 14 April 2021).

## Supplementary Materials

The following supporting information can be downloaded at: https://www.mdpi.com/article/10.3390/ijerph19031531/s1, Figure S1:Volunteering, mental distress and deprivation, Figure S2: Volunteering, quality of life and deprivation, Table S1: Fixed effects analysis controlling for age only, Table S2: Fixed effects analysis predicting the associations between volunteering and mental distress (GHQ-12): volunteered at least once a month vs. less, Table S3: Fixed effects analysis predicting the associations between volunteering and health-related quality of life (SF-12): volunteered at least once a month vs. less, Table S4: Fixed effects models interacting with index of multiple deprivation (IMD) 10% most deprived areas, Table S5: Fixed effects models interacting with index of multiple deprivation (IMD) rank.
