# Supplementary material for: Relationships between Volunteering, Neighbourhood Deprivation and Mental Wellbeing across Four British Birth Cohorts: Evidence from 10 Years of the UK Household Longitudinal Study
Source: Int J Environ Res Public Health. 2022 Jan 29;19(3):1531. doi: 10.3390/ijerph19031531 (PMC8835177; doi:10.3390/ijerph19031531)
Supplement: Supplementary file 1 [file ijerph-19-01531-s001.zip › ijerph-1506756-supplementary.pdf]

Table S1 Fixed effects analysis controlling for age only

	Mental distress (GHQ12)			Health-related quality of life (SF12)			Number of obs	Number of groups
	Coef	95%CI	p-value	Coef	95%CI	p-value		
Whole sample	-0.01	-0.02, 0.00	0.187	**0.02**	**0.01, 0.04**	**0.009**	51,206	10,989
Pre-1945 (born before 1945)	-0.02	-0.05, 0.00	0.080	**0.05**	**0.01, 0.08**	**0.011**	7,351	1,491
Baby Boomers (born in 1945-64)	**-0.03**	**-0.05, -0.01**	**0.005**	**0.03**	**0.01, 0.05**	**0.005**	21,809	4,431
Gen X (born in 1965-79)	0.02	-0.01, 0.04	0.195	-0.01	-0.04, 0.02	0.493	13,256	2,702
Millennials (born in 1980 or after)	0.00	-0.04, 0.04	0.949	0.02	-0.02, 0.06	0.327	8,790	2,365

Notes: All models controlled all time-invariant variables. Bold values denote statistical significance at the $p < 0.05$ level.

Table S2 Fixed effects analysis predicting the associations between volunteering and mental distress (GHQ-12): volunteered at least once a month vs less

	Model 1 Volunteering			Model 2 = Model 1 + demography & SEP			Model 3 = Model 2 + IMD			Number of obs	Number of groups
	Coef	95%CI	p-value	Coef	95%CI	p-value	Coef	95%CI	p-value		
Whole sample	**-0.02**	**-0.03, -0.00**	**0.043**	**-0.02**	**-0.03, -0.00**	**0.028**	**-0.02**	**-0.03, -0.00**	**0.029**	51,206	10,989
Pre-1945 (born before 1945)	**-0.03**	**-0.06, -0.00**	**0.038**	-0.02	-0.05, 0.00	0.094	-0.02	-0.05, 0.00	0.094	7,351	1,491
Baby Boomers (born in 1945-64)	**-0.02**	**-0.05, -0.00**	**0.028**	**-0.03**	**-0.05, -0.00**	**0.016**	**-0.03**	**-0.05, -0.00**	**0.016**	21,809	4,431
Gen X (born in 1965-79)	0.01	-0.02, 0.04	0.500	0.00	-0.02, 0.03	0.786	0.00	-0.02, 0.03	0.789	13,256	2,702
Millennials (born in 1980 or after)	-0.02	-0.07, 0.02	0.316	-0.02	-0.06, 0.02	0.379	-0.02	-0.06, 0.02	0.389	8,790	2,365

Notes: All models controlled all time-invariant variables. Model 1 additionally included volunteering engagement. Model 2 = Model 1 + demography (age, partnership status, whether or not living with parents, whether or not living with children, number of close friends, and long-standing illness or impairment) & SEP (education levels, employment statues, and individual monthly income). Model 3 = Model 2 + IMD (whether or not living in the 20% most deprived areas). Bold values denote statistical significance at the $p < 0.05$ level.

Table S3 Fixed effects analysis predicting the associations between volunteering and health-related quality of life (SF-12): volunteered at least once a month vs less

	Model 1 Volunteering			Model 2 = Model 1 + demography & SEP			Model 3 = Model 2 + IMD			Number of obs	Number of groups
	Coef	95%CI	p-value	Coef	95%CI	p-value	Coef	95%CI	p-value		
Whole sample	**0.02**	**0.00, 0.04**	**0.022**	**0.02**	**0.00, 0.04**	**0.039**	**0.02**	**0.00, 0.04**	**0.039**	51,206	10,989
Pre-1945 (born before 1945)	**0.09**	**0.05, 0.13**	**0.000**	**0.05**	**0.01, 0.08**	**0.011**	**0.05**	**0.01, 0.08**	**0.011**	7,351	1,491
Baby Boomers (born in 1945-64)	**0.02**	**0.00,0.05**	**0.039**	**0.03**	**0.01, 0.05**	**0.011**	**0.03**	**0.01, 0.05**	**0.011**	21,809	4,431
Gen X (born in 1965-79)	-0.02	-0.06, 0.02	0.313	-0.01	-0.05, 0.02	0.491	-0.01	-0.05, 0.02	0.484	13,256	2,702
Millennials (born in 1980 or after)	0.02	-0.03, 0.07	0.473	0.01	-0.03, 0.05	0.668	0.01	-0.04, 0.05	0.674	8,790	2,365

Notes: All models controlled all time-invariant variables. Model 1 additionally included volunteering engagement. Model 2 = Model 1 + demography (age, partnership status, whether or not living with parents, whether or not living with children, number of close friends, and long-standing illness or impairment) & SEP (education levels, employment statues, and individual monthly income). Model 3 = Model 2 + IMD (whether or not living in the 20% most deprived areas). Bold values denote statistical significance at the $p < 0.05$ level.

Table S4 Fixed effects models interacting with index of multiple deprivation (IMD) 10% most deprived areas

	Mental distress (GHQ 12)			Health-related quality of life (SF-12)			Number of obs	Number of groups
	Coef	95%CI	p-value	Coef	95%CI	p-value		
Whole sample							51,206	10,989
Volunteering	-0.01	-0.02, 0.01	0.232	**0.02**	**0.01, 0.04**	**0.004**		
IMD 10% most deprived	-0.05	-0.10, 0.01	0.090	0.03	-0.05, 0.11	0.445		
Volunteering*IMD 10% most deprived	-0.05	-0.14, 0.03	0.202	0.06	-0.04, 0.16	0.245		
Pre-1945 (born before 1945)							7,351	1,491
Volunteering	-0.02	-0.05, 0.00	0.068	**0.05**	**0.11, 0.08**	**0.010**		
IMD 10% most deprived	-0.10	-0.27, 0.07	0.237	-0.10	-0.45, 0.25	0.580		
Volunteering*IMD 10% most deprived	0.03	-0.08, 0.15	0.566	-0.02	-0.24, 0.20	0.863		
Baby Boomers (born in 1945-64)							21,809	4,431
Volunteering	**-0.03**	**-0.05, -0.01**	**0.008**	**0.03**	**0.01, 0.05**	**0.003**		
IMD 10% most deprived	0.04	-0.08, 0.15	0.526	-0.09	-0.24, 0.06	0.257		
Volunteering*IMD 10% most deprived	-0.10	-0.25, 0.06	0.227	0.09	-0.05, 0.23	0.203		
Gen X (born in 1965-79)							13,256	2,702
Volunteering	0.01	-0.01, 0.04	0.379	0.00	-0.03, 0.03	0.884		
IMD 10% most deprived	-0.01	-0.09, 0.07	0.763	**0.11**	**0.00, 0.21**	**0.045**		
Volunteering*IMD 10% most deprived	0.00	-0.14, 0.14	0.980	-0.09	-0.18, 0.01	0.081		
Millennials (born in 1980 or after)							8,790	2,365
Volunteering	0.00	-0.03, 0.04	0.796	0.01	-0.02, 0.05	0.462		
IMD 10% most deprived	-0.06	-0.14, 0.02	0.155	0.02	-0.09, 0.14	0.672		
Volunteering*IMD 10% most deprived	-0.09	-0.23, 0.05	0.219	0.14	-0.05, 0.33	0.157		

Notes: All models controlled all variables shown in the in-text analysis. Bold values denote statistical significance at the $p < 0.05$ level.

Figure S1: Volunteering, mental distress and deprivation

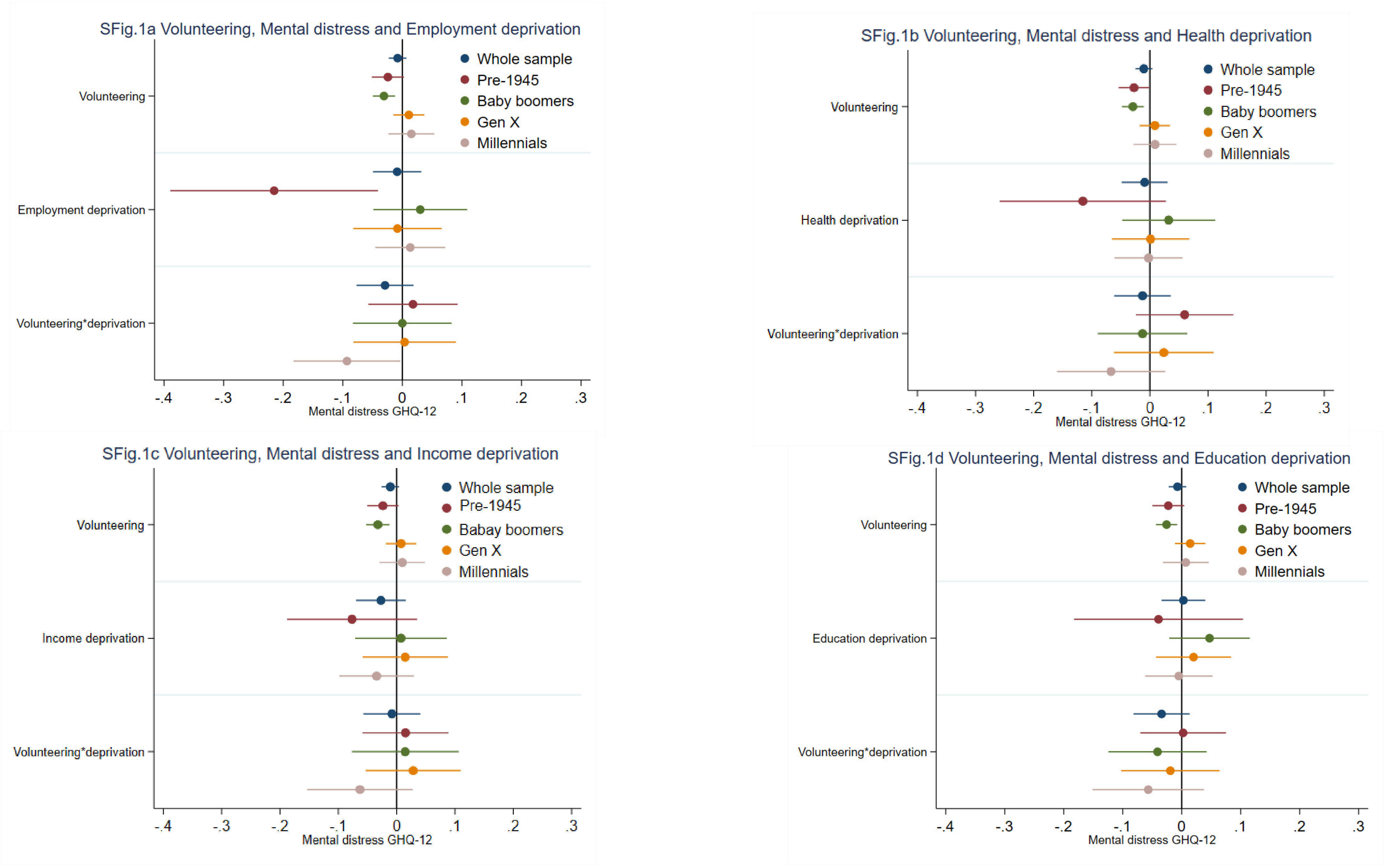

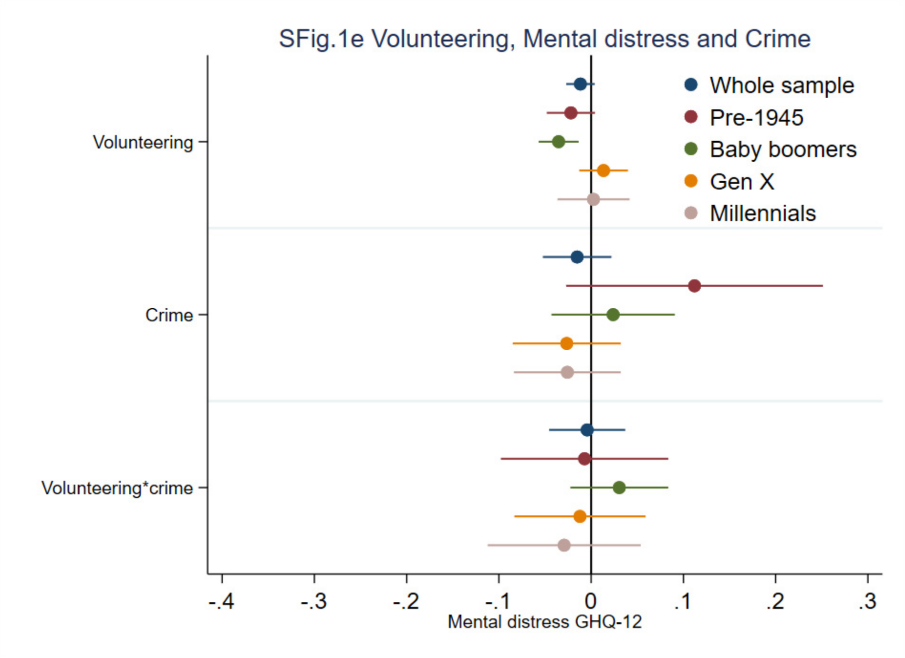
SFig.1e Volunteering, Mental distress and Crime
Whole sample
Pre-1945
Baby boomers
Gen X
Millennials
Volunteering
Crime
Volunteering*crime
-.4
-.3
-.2
-.1
0
.1
.2
.3
Mental distress GHQ-12

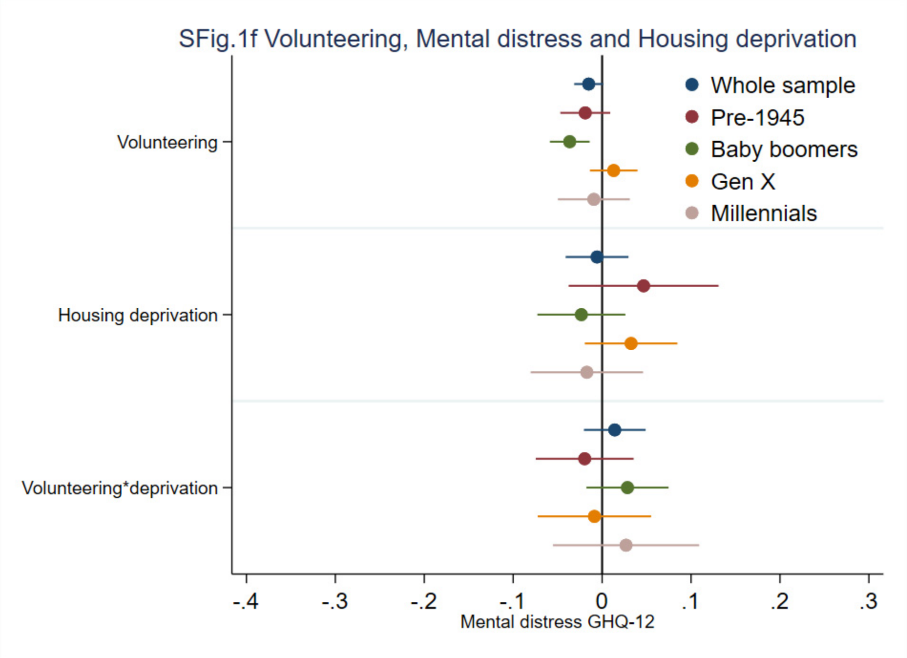
SFig.1f Volunteering, Mental distress and Housing deprivation
Whole sample
Pre-1945
Baby boomers
Gen X
Millennials
Volunteering
Housing deprivation
Volunteering*deprivation
-.4
-.3
-.2
-.1
0
.1
.2
.3
Mental distress GHQ-12

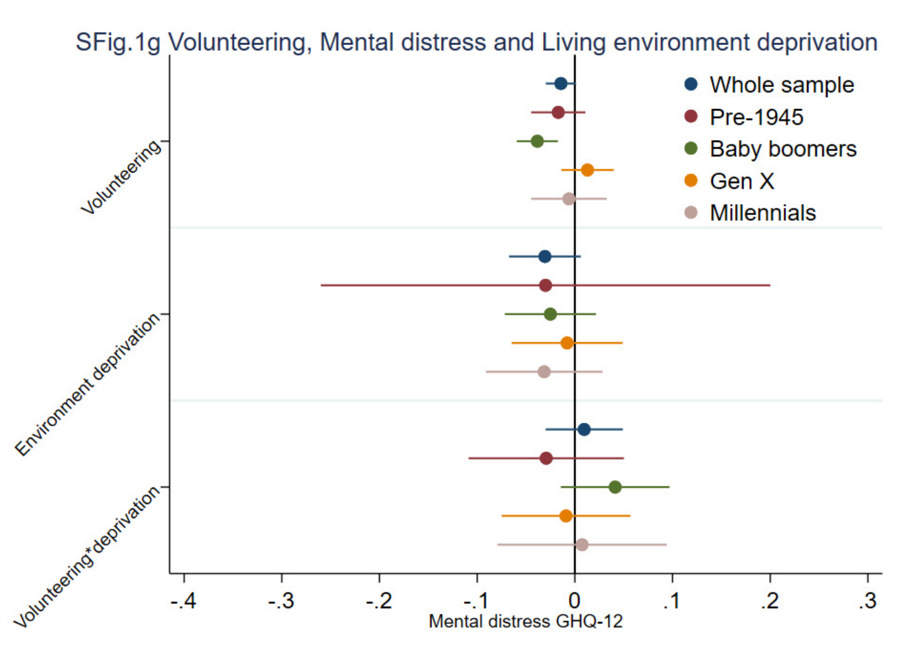
SFig.1g Volunteering, Mental distress and Living environment deprivation
Whole sample
Pre-1945
Baby boomers
Gen X
Millennials
Volunteering
Environment deprivation
Volunteering*deprivation
-.4
-.3
-.2
-.1
0
.1
.2
.3
Mental distress GHQ-12

Figure S2: Volunteering, quality of life and deprivation

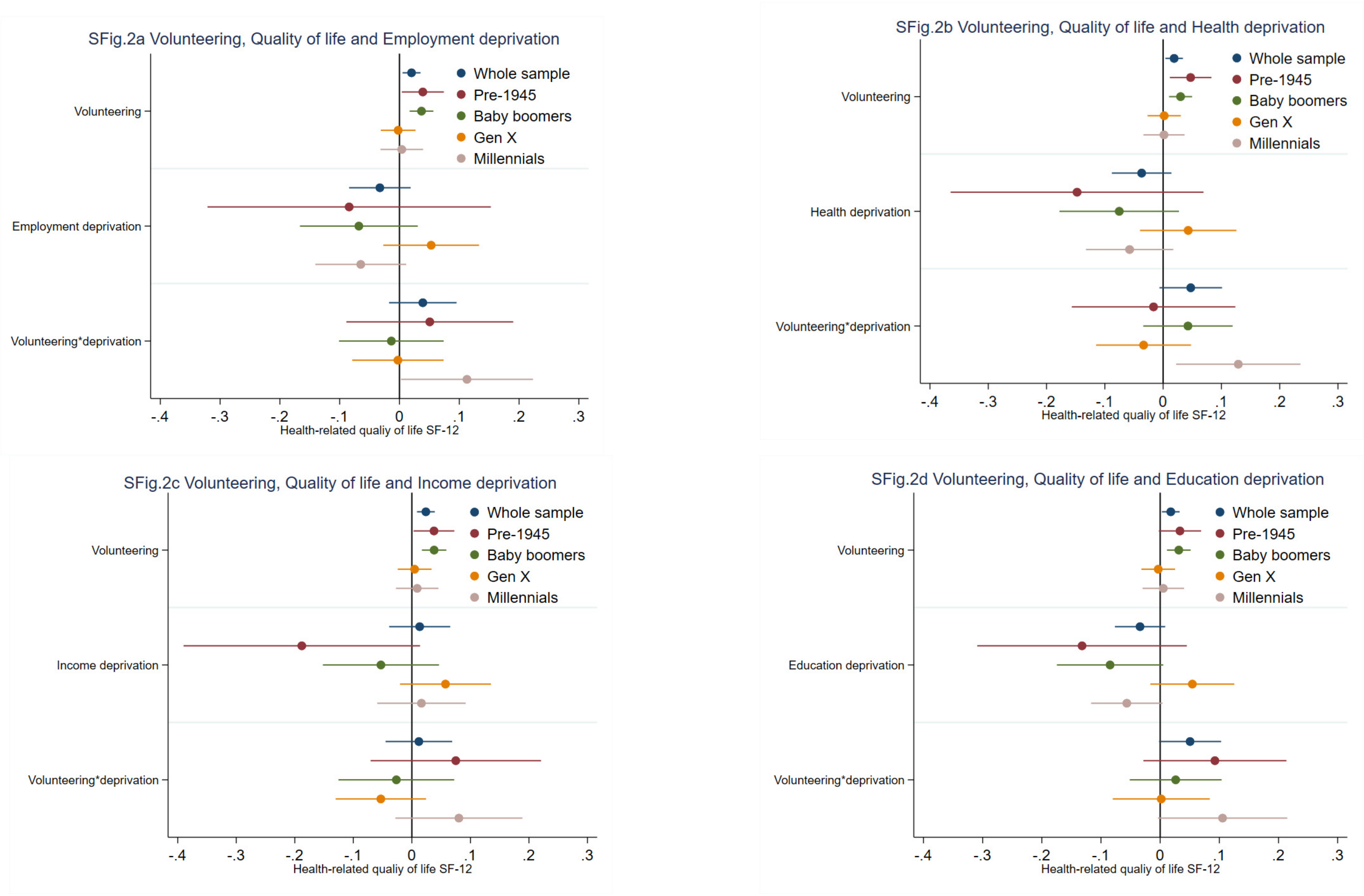

SFig.2e Volunteering, Quality of life and Crime

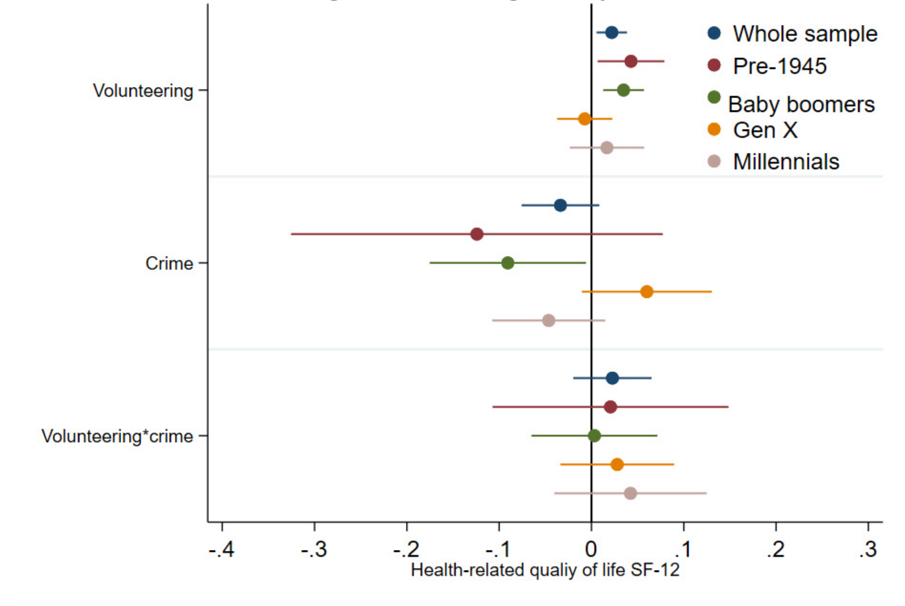

SFig.2f Volunteering, Quality of life and Housing deprivation

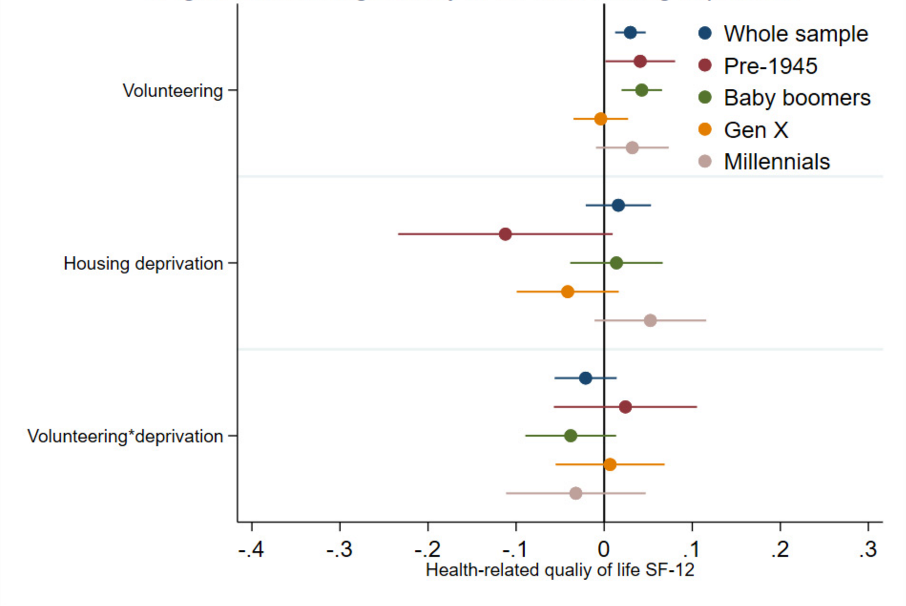

SFig.2g Volunteering, Quality of life and Living environment deprivation

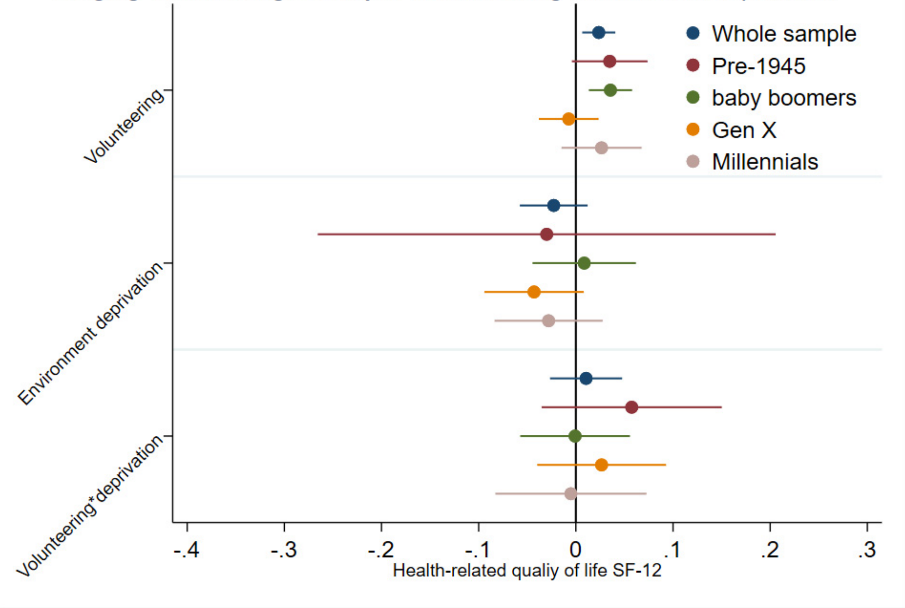

Table S5 Fixed effects models interacting with index of multiple deprivation (IMD) rank

	Mental distress (GHQ 12)			Health-related quality of life (SF-12)			Number of obs	Number of groups
	Coef	95%CI	p-value	Coef	95%CI	p-value		
Whole sample							51,206	10,989
Volunteering	-0.02	-0.06, 0.01	0.226	**0.06**	**0.02, 0.10**	**0.005**		
IMD rank	0.00	-0.00, 0.00	0.452	0.00	-0.00, 0.00	0.172		
Volunteering*IMD rank	0.00	-0.00, 0.00	0.479	-0.00	-0.00, 0.00	0.053		
Pre-1945 (born before 1945)							7,351	1,491
Volunteering	-0.02	-0.08, 0.04	0.461	0.09	-0.01, 0.18	0.082		
IMD rank	0.00	-0.00, 0.00	0.368	0.00	-0.00, 0.00	0.056		
Volunteering*IMD rank	0.00	-0.00, 0.00	0.987	-0.00	-0.00, 0.00	0.348		
Baby Boomers (born in 1945-64)							21,809	4,431
Volunteering	-0.02	-0.08, 0.04	0.442	0.03	-0.03, 0.09	0.378		
IMD rank	-0.00	-0.00, 0.00	0.221	0.00	-0.00, 0.00	0.205		
Volunteering*IMD rank	-0.00	-0.00, 0.00	0.770	0.00	-0.00, 0.00	0.754		
Gen X (born in 1965-79)							13,256	2,702
Volunteering	-0.01	-0.07, 0.04	0.614	0.02	-0.04, 0.08	0.574		
IMD rank	0.00	-0.00, 0.00	0.889	-0.00	-0.00, 0.00	0.199		
Volunteering*IMD rank	0.00	-0.00, 0.00	0.286	-0.00	-0.00, 0.00	0.405		
Millennials (born in 1980 or after)							8,790	2,365
Volunteering	-0.03	-0.11, 0.04	0.407	**0.11**	**0.03, 0.19**	**0.008**		
IMD rank	0.00	-0.00, 0.00	0.672	0.00	-0.00, 0.00	0.052		
Volunteering*IMD rank	0.00	-0.00, 0.00	0.353	**-0.00**	**-0.00, -0.00**	**0.011**		

Notes: Notes: All models controlled all variables shown in the in-text analysis. Bold values denote statistical significance at the $p < 0.05$ level.
